# Polyploidy Promotes Hypertranscription, Apoptosis Resistance, and Ciliogenesis in Cancer Cells and Mesenchymal Stem Cells of Various Origins: Comparative Transcriptome In Silico Study

**DOI:** 10.3390/ijms25084185

**Published:** 2024-04-10

**Authors:** Olga V. Anatskaya, Alexander E. Vinogradov

**Affiliations:** Institute of Cytology Russian Academy of Sciences, 194064 St. Petersburg, Russia; aevin@incras.ru

**Keywords:** cancer cell, mesenchymal stem cell, oncologic safety, therapeutic properties, circadian clock, polyploidy, DNA damage, chromatin opening, NUA4/TIP60, histone acetylation, cilia, centrosome, hypertranscription

## Abstract

Mesenchymal stem cells (MSC) attract an increasing amount of attention due to their unique therapeutic properties. Yet, MSC can undergo undesirable genetic and epigenetic changes during their propagation in vitro. In this study, we investigated whether polyploidy can compromise MSC oncological safety and therapeutic properties. For this purpose, we compared the impact of polyploidy on the transcriptome of cancer cells and MSC of various origins (bone marrow, placenta, and heart). First, we identified genes that are consistently ploidy-induced or ploidy-repressed through all comparisons. Then, we selected the master regulators using the protein interaction enrichment analysis (PIEA). The obtained ploidy-related gene signatures were verified using the data gained from polyploid and diploid populations of early cardiomyocytes (CARD) originating from iPSC. The multistep bioinformatic analysis applied to the cancer cells, MSC, and CARD indicated that polyploidy plays a pivotal role in driving the cell into hypertranscription. It was evident from the upregulation of gene modules implicated in housekeeping functions, stemness, unicellularity, DNA repair, and chromatin opening by means of histone acetylation operating via DNA damage associated with the NUA4/TIP60 complex. These features were complemented by the activation of the pathways implicated in centrosome maintenance and ciliogenesis and by the impairment of the pathways related to apoptosis, the circadian clock, and immunity. Overall, our findings suggest that, although polyploidy does not induce oncologic transformation of MSC, it might compromise their therapeutic properties because of global epigenetic changes and alterations in fundamental biological processes. The obtained results can contribute to the development and implementation of approaches enhancing the therapeutic properties of MSC by removing polyploid cells from the cell population.

## 1. Introduction

Mesenchymal stem cells (MSC), commonly referred to as “adult stem cells”, are multipotent stromal cells of diverse origins. The highest concentrations of these cells are found in the bone marrow, umbilical cord blood, placenta, endometrium, and adipose tissue [1,2]. According to the concept of the perivascular origin of MSC, these cells are ubiquitously present in almost all vascularized tissues [3]. The remarkable properties of MSC include the ability to self-renew and multipotently differentiate into various cell types such as osteoblasts, chondrocytes, myocytes, and adipocytes [4,5,6,7]. In addition, they exhibit the production of a variety of beneficial growth factors and cytokines [8,9], which distinguishes them from other cell types [5].

Attracting attention of both scientists and clinicians, MSC have gained increasing importance due to their unique properties that make them exceptionally valuable for medical applications. Noteworthy, among these qualities are their ease of isolation and cultivation, plasticity, and their inherent tropism for damaged areas—a phenomenon known as homing [10]. In addition, MSC are effective modulators of inflammatory responses, promoting processes of tissue repair, healing or regeneration through the production of numerous mediators, cytokines, chemokines, and signaling molecules [11].

While exhibiting anti-inflammatory, anti-apoptotic, and antibacterial effects in damaged tissues, MSC also have the ability to activate other resident stem cells and stimulate neo angiogenesis [10]. The clinical picture reflects the growing importance of MSC: more than 1450 clinical trials of MSC involving several thousand patients have been registered (www.clinicaltrials.gov assessed on 14 December 2023). Despite the ongoing evolution of the scientific understanding of stem cell tissue differentiation, transplantation, and integration [12], MSC have already become a reality in clinical settings due to their enormous therapeutic potential [3]. The natural scarcity of MSC in adult tissues requires extensive ex vivo culture to achieve significant numbers of cells for clinical use [5]. However, this in vitro expansion process creates problems that contribute to the manifestations of aging, genetic instability, and accelerated polyploidization, which leads to the accumulation of additional genomes in cells of both human and murine origin [3,13]. Although aging and genetic instability are generally accepted criteria for treatment failure, the impact of polyploidy in this context remains largely unexplored [1,2].

The phenomenon of polyploidy Implies complete duplication of the entire genome [14,15]. Unlike aneuploidy and polyteny, polyploidy is not accompanied by large-scale chromosomal rearrangements, including deletions, insertions, and translocations, because it maintains gene dosage balance [14,16]. Apparently, that is why polyploidy has only recently started to attract attention. At the same time, polyploidy can promote gene expression deregulation via epigenetic changes caused by chromatin opening originating from an altered nuclear surface to volume ratio and other rearrangements of chromatin architecture [17]. Currently, polyploidy is considered as a prerequisite of global DNA instability and aneuploidy [18,19,20]. It is also important to note that recent investigations revealed a tight association between polyploidy and genome instability that appears during DNA replication in the first S phase following induction of tetraploidy [18,21]. It was also established that polyploidy in proliferating cells can provoke malignancy via unscheduled polyploidy [18,21,22]. Moreover, polyploidy is a prevailing large-scale epigenetic defect in human cancer, contributing significantly to the genesis of approximately 30–40% of all solid tumors, often manifesting as early events in tumorigenesis [18,22,23,24,25,26,27,28]. 

Currently, the impact of polyploidy on the oncological safety of MSCs remains largely unexplored. At the same time, there is evidence that polyploidy can enhance signaling pathways related to stemness, morphogenesis, growth, and survival that are able to promote carcinogenesis [29]. Thus, in adipose tissue, polyploid MSC show elevated levels of specific stemness factors compared to their diploid counterparts, including Krueppel-like Factor 4 (KLF4) and secreted growth factors such as Insulin-like Growth Factor 1 (IGF1), reflecting a promotion of the embryonic phenotype and activation of signaling pathways related to growth processes [30]. Accordingly, the investigations of murine heart interstitial cells revealed that tetraploid cells exhibit a higher proliferative potential than diploid counterparts, demonstrating an ability to evade senescence associated with DNA instability and endomitosis [31]. In essence, these findings collectively imply that polyploidy has the capacity to modify the biological properties of MSC and the composition of their secreted paracrine factors, potentially compromising the oncological safety of both the MSC themselves and the cells they interact with.

In this study, we focused on the identification of the common features of polyploidy, primarily related to the oncogenic potential in cells with valuable therapeutic properties, such as MSC. The investigation of how polyploidy affects oncological safety of MSC, will enables us to further improve their clinical properties and will lead to a better understanding of ploidy-related mechanisms of gene regulation. For this purpose, we utilized an extensive comparative transcriptome analysis of the publicly accessible gene expression datasets containing evidence of ploidy-associated gene expression changes in cancer cells and in MSC. We used this comparative approach because of its ability to reveal fundamental, evolutionarily conserved traits in transcriptome and gene regulatory networks that are common in cells with different specialization and proliferation abilities [32]. 

The extensive search of databases with appropriate quality yielded four databases obtained with MSC of various origins (i.e., MSC of mouse bone marrow, human and mouse placenta, and mouse interstitial cardiac cells) [31,33,34,35]. In all these databases, the authors presented and compared mRNA seq data for isolated and sorted tetraploid and diploid cells. Moreover, in all studies the authors declared that they focused only on polyploidy—related features. For cancer cells, we used the comprehensive database integrating sequencing data from roughly 10,000 primary human cancer samples and essentiality data from approximately 600 cancer cell lines [22]. Here we identified and investigated the common effects of polyploidy, i.e., the effects that are evolutionarily conserved in cancer cells and in MSC. To find these fundamental effects, we revealed common manifestations of polyploidy by analyzing consistently ploidy up- or downregulated genes through all databases and all comparisons. 

To validate the applicability of the identified ploidy-associated gene signatures to other clinically relevant cell types, we compared the obtained results with the mRNA sequencing data related to cardiac progenitor cells (CP) and young cardiomyocytes (CARD) obtained from the iPSC of the patients diagnosed with hypoplastic left ventricle (HPLV) syndrome and of healthy people [36]. We choose this particular database because HPLV is associated with a dramatic hyperpolyploidization of both CP and CARD cell types [36]. To our knowledge, this study is the only one where the authors carefully investigated changes in both the transcriptome and the ploidy of cardiomyocytes and their progenitors influenced by HPLV. In this paper, we focused only on the consensus ploidy-deregulated genes whose expression changed similarly in all compared cell types. 

Overall, our data revealed no link between features of oncogenic transformation and polyploidy in MSC. At the same time, we found that polyploidy induces a dramatic reorganization of basic biological properties and therefore can alter therapeutic properties of clinically relevant MSC, CP, and CARD. Specifically, our results identified a functionally cohesive picture of ploidy-related changes that are coordinated by the activation of the NUA4/Tip60 chromatin opening complex operating via histone acetylation. 

## 2. Results

### 2.1. Characterization of Ploidy Regulated Gene Sets

The analysis of the consensus ploidy up- and downregulated gene sets was performed using two different approaches. In the first approach, we investigated the complete sets of 358 upregulated genes and of 425 downregulated genes (Supplementary Tables S1 and S2). In the second approach, we focused only on the essential regulators (hub genes) identified by the gene-centered protein interaction enrichment analysis (PIEA) [37]. This procedure yielded 113 ploidy-induced and 105 ploidy-inhibited essential regulators demonstrating consistent ploidy-dependent expression (Supplementary Tables S3 and S4). We applied this approach because it can distinguish between the genes that really drive disease (i.e., hub regulators) and those that are just associated with the disease but do not play an essential role [38,39,40].

Below we provide a detailed functional description of the most important groups of the enriched gene modules that were revealed by the Metascape and the collection of gene signature analysis (see methods, please). We also paid attention to molecular complexes identified by the MCODE clustering algorithm implemented in Metascape [41]. In addition, we analyzed gene composition for essential pathways with unclear functions but implicated in cancer and cell activation using the String server [42]. 

### 2.2. Gene Modules Upregulated by Polyploidy Reveal Global Transcriptome Activation

#### 2.2.1. Polyploidy Promotes Housekeeping Functions

**Figure 1:**
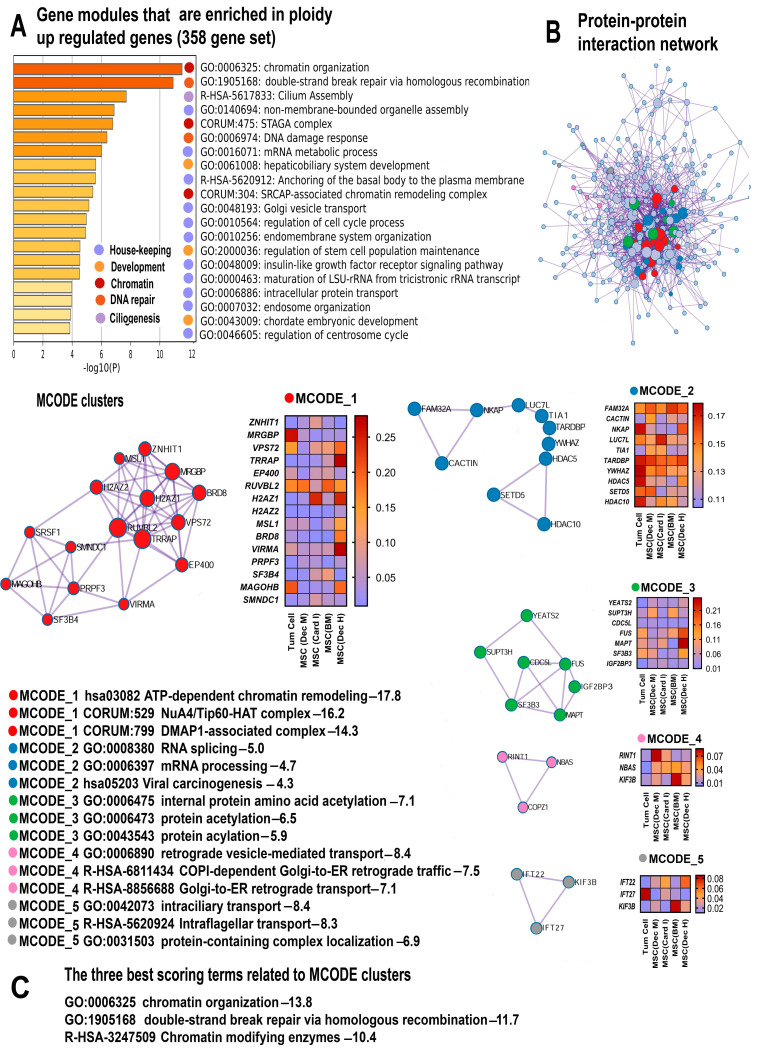
The enrichment of polyploidy-induced genes from the 358 gene set. (**A**) Bar graph of enriched terms related to gene modules and processes. The statistical significance of enrichment is shown on the X-axis (−log10 (*p*)). (**B**) Protein interaction network and MCODE components (densely connected network components) identified in the gene list. The network and MCODE components were constructed on the basis of physical interactions taken from the String server (physical score > 0.4). The coding by colored circles indicates the results of the MCODE component pathway and process enrichment analysis. (**C**) The three best-scoring terms related to the MCODE components. The figure illustrates the main effects of polyploidy, including the chromatin opening via ATP dependent remodeling and the NUA4/Tip60 histone acetylating complex. It also demonstrates the boosting of housekeeping processes, development, DNA repair, and ciliogenesis associated with the boosting of the centrosome cycle (**A**,**B**).

**Figure 2:**
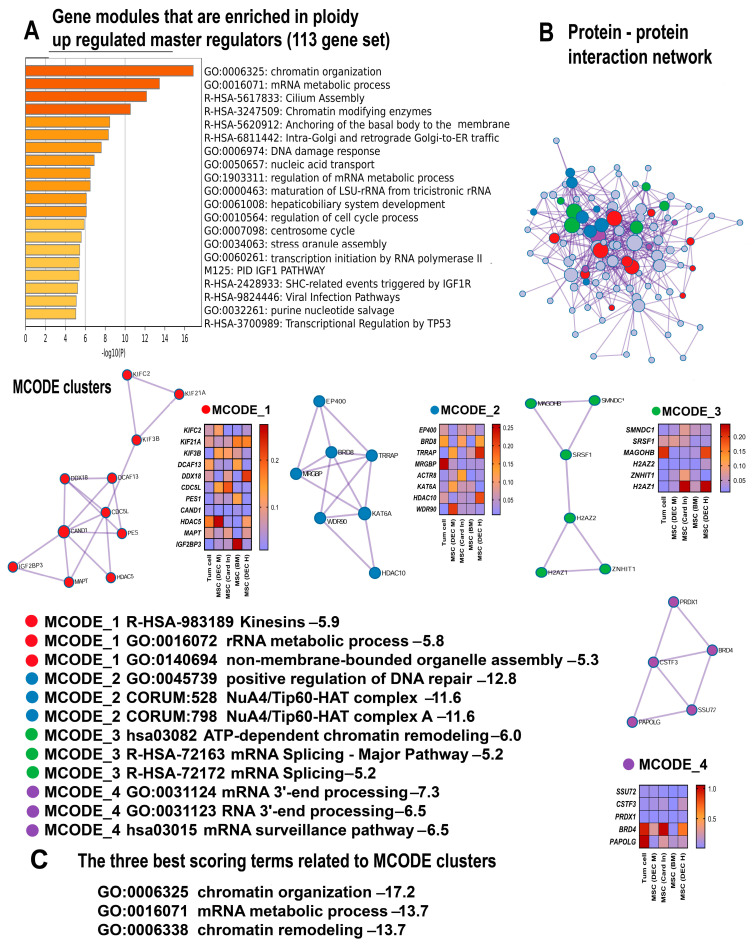
The enrichment of polyploidy-induced genes from the 113 gene set including master regulators. (**A**) Bar graph of enriched terms related to gene modules and processes. The statistical significance of enrichment is shown on the X-axis (−log10 (*p*)). (**B**) Protein interaction network and MCODE components (densely connected network components) identified in the gene list. The network and MCODE components were constructed on the basis of physical interactions taken from the String server (physical score > 0.4). The coding by colored circles indicates the results of the MCODE component pathway and process enrichment analysis. (**C**) The three best-scoring terms related to the MCODE components. This figure illustrates a good concordance between the data obtained for all 358 genes and for the master regulators. This is clearly seen for general chromatin remodeling (**A**–**C**) and chromatin remodeling associated with the NuA4/Tip60 histone acetylating complex. It also uncovers the activation of housekeeping and developmental processes, DNA repair (**A**,**B**), ciliogenesis, and the centrosome cycle (**A**).

The pathway enrichment analysis uncovered a comprehensive picture indicating that polyploidy enhances the transcriptome. This is evident from the features of hypertranscription, such as the activation of housekeeping functions and the upregulation of gene modules containing evolutionarily conserved essential genes that are necessary for cell survival [43]. The functional enrichment analysis of 358 ploidy-upregulated genes and 113 upregulated master regulators (Supplementary Tables S1 and S2), using the collection of gene signatures, revealed the highly significant upregulation of “housekeeping genes”, “top 10% AT3 SW-degenerate synonymous sites”, and “top 10% selection-favored in primates vs. rodents” (Table 1). These gene signatures include the vital human genes that are under the strongest purifying selection [37,44]. The induction of these signatures points to the activation of cellular processes especially important for cell survival. Similarly, the “3rd PIN cluster” is associated with housekeeping cellular functions adapted for multicellular organization [45]. Furthermore, the Metascape analysis, MCODE clustering, and the enrichment of gene signatures identified a pronounced upregulation of gene modules associated with housekeeping functions including ribosomal RNA maturation, cell cycle regulation, cellular transport, organelle assembly and biogenesis, insulin-like growth factor receptor signaling pathway, and mRNA metabolism (Figure 1A,B and Figure 2A,B).

#### 2.2.2. Polyploidy Reawakens Programs of Unicellularity and Stemness

Another important feature of the global transcriptome activation is the reawakening of genetic programs that are typically dormant in differentiated cells [43]. These programs trigger a regression to fundamental traits associated with unicellularity, early embryonic development, and cellular pluripotency [51,52]. The phylostratigraphy analysis indicated that both sets of the upregulated genes (i.e., the 358 genes and 113 master regulators, Supplementary Tables S1 and S2) are enriched in the genes of unicellular origin, i.e., belonging to the first two evolutionary phylostrata (Prokaryota-unicellular Eukaryota) according to phylostratigraphy [48,49], as well as to the unicellular genes in the giant unicellular cluster of interactome according to [44] (Table 1). Another evidence of unicellularity is the enrichment of the 358 gene set in a signature with the conserved genes shared between human and yeast “Human-yeast 1:1 orthologs (Ensembl)” (Table 1). This result is in good agreement with previous data uncovering a tight association between polyploidy and ancient evolutionary programs of unicellularity [19,28,53,54,55]. 

The manifestations of stemness include the Metascape gene pathways “chordate embryonic development”, “regulation of stem cell population maintenance” (Figure 1A) and “hepatobiliary embryonic development”, (Figure 1A and Figure 2A). Accordingly, the enriched signatures include the pathways related to Myc signaling (“Myc_targets” and “c-Myc pathway”) (Table 1). These pathways confirm that polyploidy promotes stemness because c-Myc is one of the Yamanaki factors implicated in pluripotency maintenance [56,57,58,59,60]. 

#### 2.2.3. Polyploidy Promotes Chromatin Opening and Activates Related Double Strand Break DNA Repair Pathways

One of the usual causes of global transcriptome activation, or hypertranscription, is the chromatin opening [43,61]. In accordance with this observation, our Metascape analysis identified the highly significant upregulation of several gene modules related to chromatin organization and epigenetics (Figure 1A–C and Figure 2A–C). Remarkably, the modules “SRCAP-associated chromatin remodeling complex” and “SAGA/STAGA complex” promote chromatin relaxation. The SRCAP complex is an ATP-dependent chromatin remodeler [62], whereas the “SAGA/STAGA complex” is a chromatin-modifying transcriptional coactivator interacting with DNA damage-binding factors and the c-Myc oncogene [63,64,65]. In accordance with this data, the enrichment analysis performed using the collection of gene signatures uncovered the upregulation of several pathways related to signaling via c-Myc and DNA damage (Table 1).

In line with these results, the MCODE clustering identified a molecular complex NuA4/Tip60-HAT related to chromatin decondensation. This complex enriches the ploidy-induced genes with a remarkable statistical significance (*p* < 10^−16^ for the 358 gene set and *p* < 10^−11^ for 115 genes, as shown in Figure 1A–C; Figure 2A–C). The NuA4/Tip60-HAT is a histone acetyltransferase/chromatin remodeling complex playing a pivotal role in chromatin relaxation and DNA repair [66]. It is also important that the NuA4/Tip60-HAT in association with the STAGA acetyltransferase complex promotes highly reliable and accurate DNA repair through the pathway of the “double strand DNA repair” executed via homologous recombination [67]. This result is particularly important in the context of adaptive strategies related to polyploidy. 

#### 2.2.4. Polyploidy Boosts Ciliogenesis and Centrosome Cycle

The Metascape analysis and the collection of gene signatures uncovered the enrichment of ploidy-upregulated genes in the gene modules related to primary cilia, transport via cilia, and the centrosome cycle. Our data found the pathways “cilium assembly”, “intraflagellar transport”, “intraciliary transport”, and “regulation of centrosome cycle” (Figure 1A and Figure 2A) and the gene modules “Anchoring of the basal body to the plasma membrane” and “Loss of proteins required for interphase microtubule organization from the centrosome” (Table 1). In accordance, the MCODE clustering revealed the molecular complexes involved in intraciliary transport and intraflagellar transport (Figure 1B,C). The simultaneous upregulation of gene modules implicated in ciliogenesis and the centrosome cycle can be explained by the fact that cilia consist of exactly the same microtubule barrels as centrioles that normally live within the centrosome [68].

### 2.3. Polyploidy Downregulates Gene Modules Related to Immunity, Apoptosis, and the Circadian Clock

#### 2.3.1. Polyploidy Downregulates Pathways Related to Cell Death and Apoptosis

**Figure 3:**
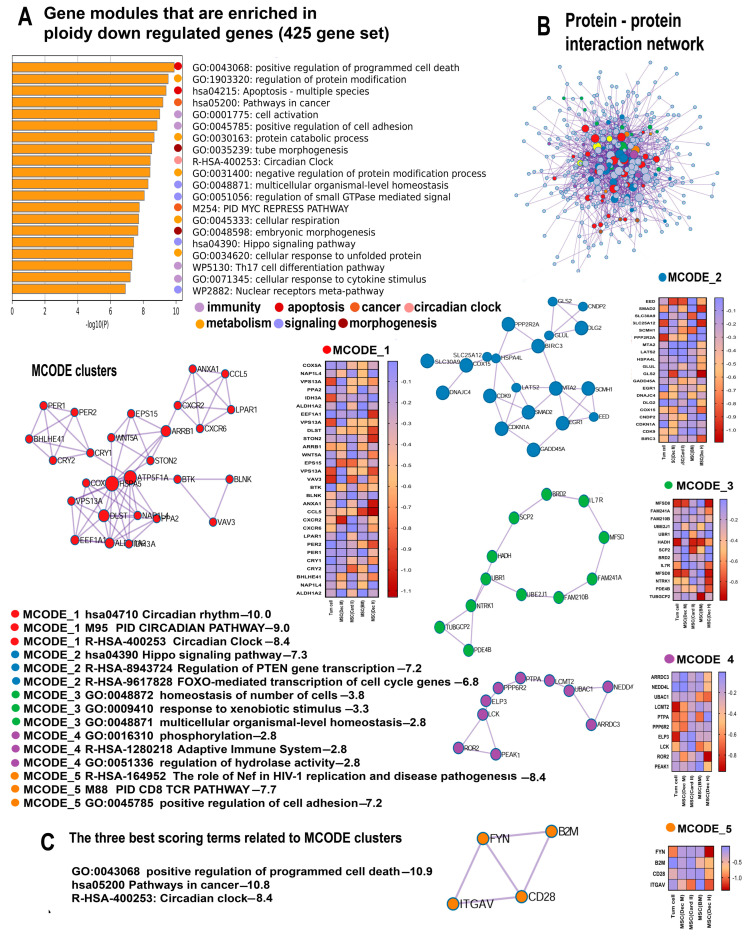
The enrichment of polyploidy-inhibited genes related to the set of 425 genes. (**A**) Bar graph of enriched terms related to gene modules and processes. The statistical significance of enrichment is shown on the X-axis (−log10 (*p*)). (**B**) Protein interaction network and MCODE components (densely connected network components) that were identified in the gene list. The network and MCODE components were constructed on the basis of physical interactions taken from the String server (physical score > 0.4). The coding by colored circles indicates the results of the MCODE component pathway and process enrichment analysis. (**C**) The three best-scoring terms related to the MCODE components. This figure illustrates the main functional features of gene modules enriching for all ploidy-inhibiting genes, including the downregulation of pathways related to the circadian clock, cell death and apoptosis (**A**–**C**), (**A**,**B**), and immunity.

**Figure 4:**
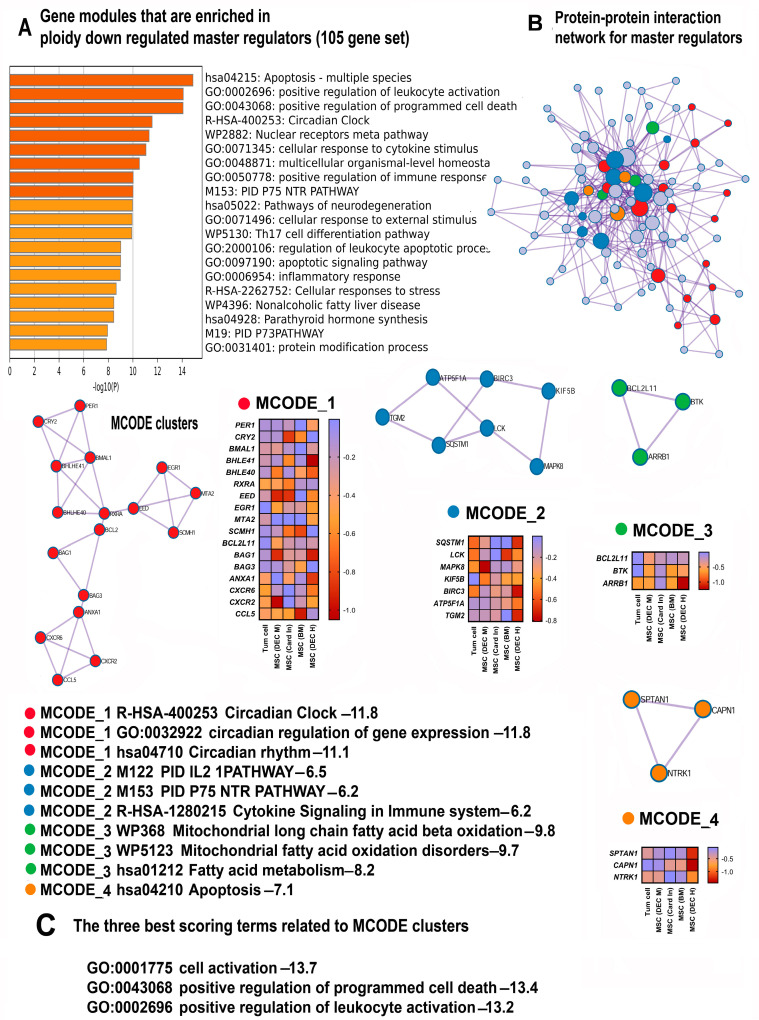
The enrichment of polyploidy-inhibited genes from the 105 gene set including master regulators. (**A**) Bar graph of enriched terms related to gene modules and processes. The statistical significance of enrichment is shown on the X-axis (−log10 (*p*)). (**B**) Protein interaction network and MCODE components (densely connected network components) that were identified in the gene list. The network and MCODE components were constructed on the basis of physical interactions taken from the String server (physical score > 0.4). The coding by colored circles indicates the results of the MCODE component pathway and process enrichment analysis. (**C**) Three best-scoring terms related to the MCODE components. This figure illustrates a good concordance between the data obtained for all 425 genes and 105 master regulators. Especially, it is seen for the gene modules implicated in apoptosis, immunity (**A**–**C**), and the circadian clock (**A**,**B**).

The most important gene module identified by the Metascape analysis that demonstrates highly significant ploidy-dependent downregulation for all genes (425 genes, Supplementary Table S3) and for master regulators (105 genes, Supplementary Table S4) is the “Pathway in cancer” (hsa05200) (Figure 3A and Figure 4A, Table 2).

**Figure 5:**
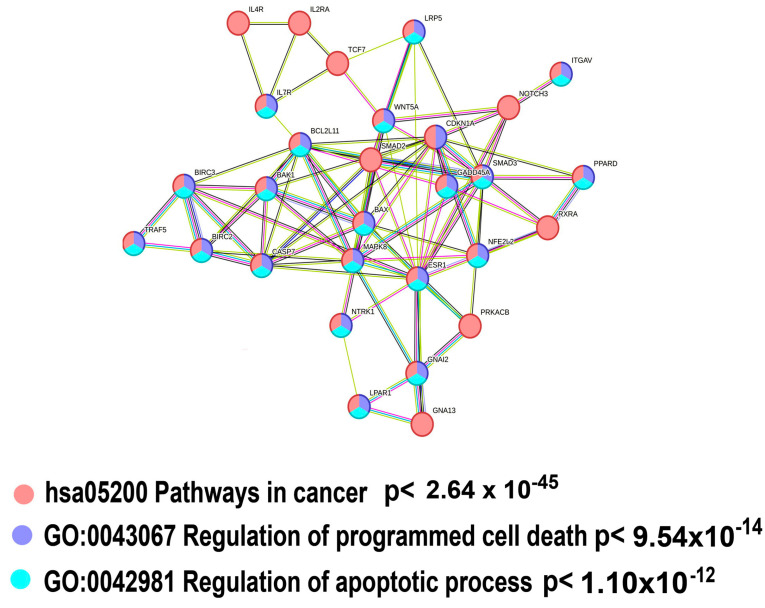
Gene composition of the gene module “Pathway in cancer “(hsa05200) enriched in ploidy-downregulated genes. It can be seen that about 70% of the pathway genes (21 out of 30) are related to the regulation of apoptosis and programmed cell death (blue and cyan labels). The protein–protein interaction network was constructed using the String Database [31] at a stringency of 0.9.

The downregulation of “Pathway in cancer” might suggest that polyploidy plays a role in tumor suppression. To investigate this suggestion, we analyzed the gene composition of this pathway using the String server (Figure 5). The results indicated that about 70% of genes encompassing “Pathways in cancer” (i.e., 20 of 28) were implicated in the regulation of cell death (“Regulation of programmed cell death” (GO:0043067)), thus pointing to the fact that the ploidy-downregulated branch of the “Pathway in cancer” module is not related to tumor suppression (but rather, exhibits an opposite effect). Our manually curated collection of gene signatures indicates that polyploidy is associated with the downregulation of tumor suppressor genes (“TSG downregulated pancancer”) (Table 2), thus confirming that polyploidy may rather activate carcinogenesis than suppress it.

In accordance with the pro-carcinogenic changes in “Pathways in cancer”, the Metascape analysis identified the suppression of two gene modules related to apoptosis and cell death: “Apoptosis, multiple species” and “Positive regulation of programmed cell death”. The pathways demonstrate a highly significant enrichment (*p* < 10^−8^ and *p* < 10^−15^ for the 425 and 108 gene sets, respectively, Figure 3A and Figure 4A). Our collection of gene signatures also confirmed the association between polyploidy and the downregulation of pro-apoptotic pathways (Table 2). Moreover, it revealed the downregulation of several pathways related to tumor suppression (Table 2).

The Metascape and the collection of gene signatures analysis indicate that the anti-apoptotic effects of polyploidy are complemented by the downregulation of the “Validated targets of C-MYC transcriptional repression” encompassing the genes that are repressed by the Myc oncogene (Figure 3A, Table 2). Thus, the effects of polyploidy found in this study are in good agreement with the previous observations underpinning the anti-apoptotic effects of polyploidy in various types of cells and the association between polyploidy and Myc oncogene induction [69,70].

The pro-survival and anti-apoptotic features of polyploidy were accompanied by the downregulation of the pathways implicated in morphogenesis and cellular adhesion, including “tube morphogenesis”, “embryonic morphogenesis”, and “positive regulation of cell adhesion” (Figure 3A). Considering the multifaceted activation of developmental, proliferative, and stemness-related pathways by polyploidy, one can conclude that the influence of polyploidy on the Morphogenesis–Survival–Death axis is very complex and reveals a bias towards cell survival. This interplay of ploidy-associated genetic events underscores the complexities in cellular regulation, shedding light on the delicate ploidy-related mechanisms managing cell fate and survival.

#### 2.3.2. Polyploidy Downregulates the Overlapping Pathways of Cell Activation and Immunity

**Figure 6:**
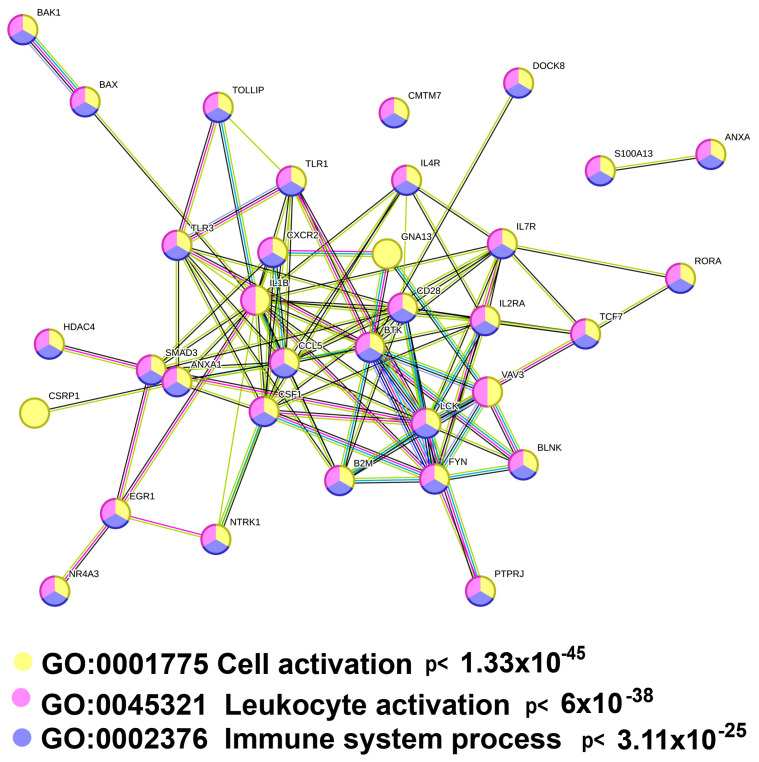
Gene composition of the gene module “Cell activation” (GO:0001775) enriched in ploidy-downregulated genes. It can be seen that about 90% of the pathway genes (31 out of 34) are related to the regulation of immunity (sky blue and purple labels). The protein–protein interaction network was constructed using the String Database [31] at a stringency of 0.9.

Our data identified one more intriguing gene module with unclear functions that is ploidy-downregulated with high significance, “Cell activation” GO:0001775 (Figure 3A). To elucidate the functions of this module, we analyzed the genes encompassed by this module with the String server [42]. The genes related to immunity prevail in this pathway comprising about 90% of all genes (Figure 6). This observation is in good agreement with the downregulation of several pathways related to immunity, including “Cellular response to cytokine stimulus”, “Regulation of canonical NF-kappa B signal transduction”, “Th17 cell differentiation pathway”, and “Regulation of leucocyte apoptotic processes” identified by both the Metascape and the collection of gene signatures (Table 2, Figure 3A–C and Figure 4A–C).

#### 2.3.3. Polyploidy Attenuates Pathways Related to the Circadian Clock

Further pathway enrichment analysis with the aid of the Metascape identified several gene modules related to rhythmic processes (Table 2, Figure 3A–C and Figure 4A–C). Employing MCODE clustering helped us to uncover a large molecular complex involved in the regulation of the circadian clock (Figure 3B and Figure 4B). Within this complex, the genes exhibited a strong significance of enrichment in the pathways related to circadian processes (*p* < 10^−10^ for all ploidy-regulated genes and *p* < 10^−11^ for master regulators). This high statistical significance underscores the biological relevance of these findings, indicating a tightly orchestrated ploidy-regulated network governing circadian rhythms. Accordingly, our collection of gene signatures further augments this result identifying the pathways linked to the regulation of rhythmic processes.

### 2.4. The Ploidy-Regulated Genes Derived from Early Cardiac Progenitors and Young Cardiomyocytes Obtained from iPS Demonstrate a Good Agreement with the Results Obtained on Cancer Cells and MSC

To verify whether the ploidy-related gene signature identified for cancer cells and MSC can be applied to other therapeutically relevant cells, we accessed the database presenting the mRNA sequencing data related to the cardiac progenitor cells (CP) and young cardiomyocytes (CARD) obtained from the iPC of the patients diagnosed with hypoplastic left ventricle syndrome and healthy people [36]. This study presented the first evidence that HPLV affects the transcriptome and increases the amount of polyploid cells in CP and in young CARD by several folds compared to healthy individuals. Therefore, the comparison of differences in gene expression in CP and young CARD in HPLV patients vs. healthy individuals with ploidy-related gene signature for cancer cells and MSC can elucidate the generality of manifestations of polyploidy in different cell types. 

The comparison of signatures representing the ploidy regulated genes in both cancer cells and MSC, with the mRNA sequencing data from CP and young CARD for HPLV patients, revealed 233 (for CP) and 171 (for CARD) matched polyploidy upregulated genes and 244 (for CP) and 183 (for CARD) matched polyploidy downregulated genes (Supplementary Tables S5–S8). 

The results of the bioinformatic analysis confirmed that some ploidy-associated features revealed in cancer cells and MSC for the upregulated genes (358 gene set, Table 1) can also be observed in CP (Supplementary Table S9) and young CARD (171 gene set, Supplementary Table S10). Thus, the Metascape analysis, the collection of gene signatures, and MCODE clustering identified chromatin rearrangements originating from ATP-dependent remodeling and histone acetylation via the Nua4/Tip60-HAT complex pointing to chromatin decompactization (Supplementary Figures S1A–C and S2A–C, Tables S9 and S10); The obtained results also revealed the features of hypertranscription, including the activation of gene modules related to housekeeping functions, embryonality, unicellularity and DNA damage response (Table 1; Figure 1A, Supplementary Figures S1A and S2A,B). In addition, as well as for MSC and cancer cells, the data for CP and young CARD confirmed the activation of gene modules related to ciliogenesis and the centrosome cycle (Supplementary Tables S9 and S10; Supplementary Figures S1 and S2).

Several gene modules that are enriched in genes from the ploidy-downregulated gene signature for MSC and cancer cells (425 gene set, Supplementary Table S3, Figure 3) are also overrepresented among common ploidy-downregulated genes for CP (244 gene set, Supplementary Table S11, Supplementary Figure S3) and young CARD (183 gene set, Supplementary Table S12, Supplementary Figure S4). For example, the collection of gene signatures, Metascape analysis and MCODE clustering identified the downregulation of molecular complexes related to rhythmic processes and circadian clocks, apoptosis, immunity, and Myc-repressed pathways (for CP only) (Supplementary Figures S3 and S4, Supplementary Tables S11 and S12). Thus, altogether, these results provide evidence for the universality of the ploidy-associated gene signature, supporting the importance of the role of genomic duplications in the epigenetic regulation of gene expression.

## 3. Discussion

### 3.1. The Analysis of Consensus Genes and Master Regulators Identified Common Features of Polyploidy across Cancer Cells and Adult Mesenchymal Stem Cells

The primary objective of this study was to investigate whether polyploidy has an impact on the oncologic safety of MSC through the epigenetic reprogramming of the transcriptome. To achieve this goal, we examined the shared effects of polyploidy on the transcriptome of cancer cells and adult MSC from various sources. We developed a sophisticated bioinformatic approach that allowed us to distinguish the meaningful signals from background noise and identify relevant biological features. Our focus was specifically on the consensus genes that were consistently up- or downregulated in all polyploid vs. diploid cells. Subsequently, we conducted the gene functional enrichment analysis using databases integrated into the Metascape server. Additionally, we utilized a manually curated collection of gene signatures to identify ploidy-related effects that may not be discerned through the Metascape platform. These effects encompassed traits such as unicellularity and multicellularity, carcinogenesis, tumor suppression, phylostratic gene shifts, and others. To validate these findings across other therapeutically relevant cell types, we compared the gene signatures associated with ploidy obtained from mesenchymal stem cells (MSC) and cancer cells to the ploidy-related transcriptomic alterations observed in CP and in the young CARD of patients with HPLV compared to healthy individuals [36].

Our analysis revealed shared characteristics among the genes upregulated and downregulated by ploidy, confirming the widespread presence of the presented gene signatures. Thus, using this comprehensive and integrative approach, we identified fundamental features associated with polyploidy, providing an understanding of how genome duplication alters cell biology. The insights gained from our findings may contribute to the development of novel strategies for manipulating polyploidization for therapeutic purposes.

### 3.2. Polyploidy Is Associated with a Hypertranscription State

The indications of polyploidy in gene modules, signatures, and molecular complexes suggest that genomic duplications play a role in transitioning a cell into a hypertranscription state. This becomes apparent through the link between polyploidy and well-established signs of hypertranscription. For instance, hypertranscription promotes pathways that support essential cellular functions, as well as programs associated with stem cells and unicellularity [43,61]. In line with these findings, our data reveal the activation of gene modules related to ribosome biogenesis, mRNA transcription, splicing, cell cycle, and other housekeeping cellular processes. Previous studies have also highlighted that polyploidy stimulates basic cellular functions, including increased nucleolar activity, expression of ribosomal RNA, and overall gene expression [17,19,20,29,69,71,72,73]. Another significant characteristic of hypertranscription is the revival of embryonic pathways that are typically dormant in normal differentiated cells [43,61,74]. In line with this, our data point to the reactivation of multiple pathways associated with multipotent stemness and unicellular evolutionary programs. The association between polyploidy, stemness, and ancient evolutionary programs was also noted for differentiated cells, cancer cells, and entire organisms in several independent studies [17,24,25,26,27,54,75,76,77,78,79,80,81]. 

The state of hypertranscription is associated with an open, permissive chromatin structure [61]. In accordance with this phenomenon, our data indicated that polyploidy induces epigenetic changes contributing to chromatin opening. This is manifested in the activation of well-known epigenetic pathways like “Epigenetic Factors” and “Chromatin Organization”, as well as three pathways promoting chromatin decompactization. The first pathway is associated with c-Myc signaling, the second involves the “SCRAP Chromatin Remodeling Complex”, and the third is linked to the “STAGA Complex”. It is well-known that enhanced signaling through c-Myc opens chromatin at low and high levels of organization via binding to E-boxes, facilitating the movement of chromatin from the nuclear periphery to the inner part, and interacting with chromatin-opening complexes [17,28]. Recently, it was discovered that Myc isoforms interact with TRRAP, which is a component of the TIP60 complex involved in ATP-dependent chromatin remodeling that opens chromatin through histone acetylation [82]. The TIP60 complex also replaces canonical histone H2A with embryonic histone H2A.Z, activating transcription and initiating programs of stemness [83,84]. In line with these data, our results indicated that ploidy promotes overexpression of histones H2AZ1 and H2AZ2 (Supplementary Table S1, Figure 1B and Figure 2B). The second pathway of chromatin remodeling, the “SCRAP-associated Chromatin Remodeling Complex”, opens chromatin through ATP-dependent remodeling. The third pathway is represented by the signaling cascade of “STAGA”, which promotes chromatin decompactization through histone acetylation [63,65]. Altogether these results are in good agreement with recent data indicating that whole genome duplication initiates chromatin conformation changes promoting chromatin relaxation [17,85,86,87]. 

One other noteworthy finding substantiating the connection between polyploidy and chromatin decompactization is the enrichment of ploidy-induced genes belonging to the molecular complex “NuA4/Tip60”. This multi-subunit complex operates as a histone acetyltransferase chromatin opener that demonstrates a remarkable conservation across species, extending from yeast to humans [67]. Functioning as an orchestrator, the NuA4/Tip60 complex plays a pivotal role in regulating cellular homeostasis, activating the double strand break DNA repair pathway, as well as safeguarding the delicate equilibrium of stem cell maintenance and renewal [88]. The involvement of the NuA4/Tip60 complex in these cellular processes underscores its significance in the choreography of cellular dynamics and genomic regulation. It is also important to note that our results are in line with the data of a recent study providing evidence that TIP60 promotes polyploidization of neonatal cardiomyocytes during normal development [89,90], thus suggesting that we identified a really fundamental signaling axis in which TIP60 mutually interacts with polyploidy via histone acetylation.

In accordance with the literature pointing to the implication of the NuA4/TIP60 complex in the double strand break (DSB) repair pathway [88], our data uncovered the ploidy-related upregulation of the pathways involved in DNA damage response and homologous recombination. This finding is in good agreement with the previous studies indicating that polyploidy promotes genome instability from the first S-phase following the induction of tetraploidy [21]. Moreover, there is evidence indicating that polyploidy activates a highly reliable system for repairing double-strand DNA breaks through homologous recombination [91]. This confirmation not only strengthens our understanding of polyploidy but also illuminates the interplay between polyploidy and increased reliability of DNA repair. 

Summarizing our data concerning the relationships between polyploidy and hypertranscription, it is important to note that all the aforementioned characteristics of polyploidy are also hallmarks of hypertranscription established by [61]. According to our study, polyploidy in cancer cells, MSC, CP, and in young CARD similarly to hypertranscription state: (1) promotes housekeeping functions; (2) is associated with a decondensed and open chromatin landscape, maintained by the activity of euchromatic ATP-dependent chromatin remodeling complexes and histone acetylation; (3) increases rRNA transcription and protein synthesis/translational machinery; (4) activates the response to double-strand DNA breaks; and (5) induces signaling factors operating via the Myc family of transcription factors, which are universal transcriptional amplifiers [17]. Moreover, recent experimental studies have shown that polyploid hepatocytes and epithelial bladder cells differ from diploid cells in having an increased level of transcription, providing direct confirmation that polyploidy creates a specific epigenetic state supporting activated transcription [61]. It is also important to note that ploidy-associated hypertranscription may increase the complexity of gene regulatory networks, which in turn confer cells plasticity and adaptability. Recent studies presented convincing evidence that genome duplication and the associated gene regulatory networks (GRN) results in an enhanced variation in signal output, thereby increasing the potential for survival during environmental upheavals [92]. These findings are in good agreement with the well-established association between polyploidy and plasticity, adaptability, and stress resistance [15,32,92,93,94,95,96,97,98,99]. The larger DNA and chromatin masses emerging in polyploid cells can stabilize the chromatin structure in the case of solvent fluctuations of the cellular microenvironment, which may also enhance stress resistance [100]. Notably, the increased complexity of regulation (probably related to acquired stress resistance) is accompanied by the decrease of cell and organ functional capacity. It was clearly shown for cardiomyocytes and hepatocytes [20,71,101,102,103,104,105]. These facts point to the potentially detrimental effects of polyploidy on cell function and, presumably, on therapeutic properties. 

### 3.3. Polyploidy Promotes Ciliogenesis and the Centrosome Cycle

The comprehensive picture of changes associated with polyploidy is complemented by the induction of gene modules implicated in the maintenance of centrosomes, primary cilia, and intraflagellar transport that are parts of a single hybrid organelle, which, depending on cell physiological status, can function either as cilia or centrosomes [106]. 

In MSC, cancer cells, CP, and young CARD, the increased activity of pathways related to centrosomes is in good agreement with previous results demonstrating the association between polyploidy and centrosome multiplication leading to DNA instability in cancer polyploid cells [107]. To our knowledge, the association between polyploidy and ciliogenesis was not documented previously. Therefore, we cannot compare our results with the data of other authors. At the same time, the activation of ciliogenesis is in good agreement with many properties of polyploid cells. Firstly, polyploidy increases cell cycle duration and impairs cell proliferative capacity [108]. These phenomena may originate from the antagonistic relationships between the cilia formation and mitosis progression [109]. Currently, all studies connecting cilia and entry to mitosis seem to be in agreement with the fact that ciliary resorption is vital to the cell cycle beginning [110]. This mutually exclusive relationship is expected because both cilia and mitotic spindles are microtubule-based and use components of the same molecular machinery [111]. Whereas the mitotic spindle is essential for cell cycle progression and cytokinesis, the cilia supposedly prevent cells from entering the cell cycle [111]. Accordingly, it was recently shown that disruption of cytokinesis (accompanying cell polyploidization) can enhance ciliogenesis [18,112]. In addition, the expression of genes encoding cell membrane proteins and cell receptors negatively correlates with cell cycle activity [113].

Secondly, the association between polyploidy and multipotent stemness, which was noted for differentiated cancer cells [76,114] as well as for MSC (this study), can be partially explained by the important role of cilia in the transmission of multipotency signaling cascades operating via WNT, NOTCH, Hippo, mTOR, Sonic Hedgehog (SHH), platelet-derived growth factor receptor-α (PDGFRα), insulin-like growth factor 1 (IGF1), and transforming growth factor-β (TGFβ) [115,116]. It is also important to note that primary cilia-dependent signaling is required for MSC proliferation and pluripotency [115,116]. Furthermore, the primary cilia might enhance manifestations of unicellularity because cilia are an evolutionarily conserved organelle that are already widespread in unicellular flagellates, for example, in Chlamidomonas [111].

Thirdly, activated ciliogenesis helps to understand the increased resistance of polyploid cells to stress, DNA damage, apoptosis, immune surveillance, and chemotherapeutic drugs [79,91,97,117,118,119]. It was recently shown that ciliogenesis activates double strand break DNA repair via the NUA4/TIP60 chromatin acetyl transferase complex mentioned above. Specifically, the cells exhibiting reduced histone acetylation due to the impairment of the NUA4/TIP60 complex demonstrate weakened repair and prolonged arrest at the G2-M checkpoint after DNA double strand breaks [111]. Ciliogenesis also protects cells from apoptosis. Thus, primary cilia stabilization may reduce the abortive cell cycle re-entry to protect injured postmitotic neurons from apoptosis [120,121]. Additionally, ciliogenesis protects cells from immunosurveillance thereby enabling them to immunoescape [121]. The overactivation of primary cilia can increase the anticancer drug resistance [122]. Altogether, these cilia-associated properties can confer polyploid cells better survival ability under stress compared to their diploid counterparts.

### 3.4. Polyploidy Impairs Signaling via the Circadian Clock

One more important feature of polyploidy identified by our study in all investigated cell types (i.e., MSC, cancer cells, CP, and CARD) is the impaired signaling of the circadian clock. This association was also previously shown for cells of plants, differentiated mammalian tissues, and tumors [53,123]. Importantly, defective circadian regulation may also partially explain stemness, retarded proliferation, and DNA damage associated with polyploidy. The circadian clock does not work appropriately in embryonic stem cells until they enter differentiation, thereby uncovering mutually exclusive relationships between the circadian clock and stemness [124]. In accordance, several genes of the circadian clock participate in the regulation of the cell cycle and DNA damage checkpoints [125,126], thereby suggesting that circadian clock disruption promotes cell cycle retardation and DNA instability in polyploid cells. 

## 4. Materials and Methods 

### 4.1. Databases

To investigate the common features in the manifestations of polyploidy in the transcriptomes of tumor cells and MSC, we used data from an integrated database of polyploid tumors and several databases for polyploid MSC. For the tumors, a set of ploidy-regulated genes was acquired from the database prepared by Quinton and co-authors [22]. This database integrates sequencing data of various types of human cancers (about 10,000 samples) and essential data from approximately 600 cancer cell lines. 

The search for databases with mRNA sequencing and microarray data for polyploid vs. diploid MSC was conducted on the NCBI website (https://www.ncbi.nlm.nih.gov/guide/all/, accessed on 14 June 2023) using the keywords “Mesenchymal stem cell AND (polyploidy OR tetraploidy OR ploidy OR whole genome duplications) AND (transcriptome, OR mRNA sequencing, OR microarray, OR NGS OR next generation sequencing)”. From all existing databases, we selected only those that were obtained on isolated diploid and polyploid MSC. 

As a result, four databases were selected for MSC studies: 

(1) In the database presenting the data for bone marrow MSC [33], the authors generated tetraploid MSC from diploid MSC by treating them with 1 mmol/L hydroxyurea (Sigma) followed by 5 mmol/L sodium butyrate. Control treatment included 24 h incubation with fresh medium before sodium butyrate treatment. Cell ploidy level was analyzed using a FACScan flow cytometer. Gene expression profile for diploid and tetraploid cells were evaluated using microarray analysis (Affymetrix, Applied Biosystems). Microarray data are available at the Gene Expression Omnibus (GEO) website, accession number GSE39410. 

(2) In the database containing the data obtained with freshly isolated cardiac interstitial cells of the mouse [31], the authors performed cell sorting by ploidy levels, using BD FACSAria II sorting and analyzed the data with FLOWJO. After sorting, tetraploid and diploid cells were subjected to single cell mRNA sequencing and transcriptome analysis. Sequence data that support the findings of this study have been deposited into GEO with the primary accession code GSE122057. 

(3) The data base related to diploid and tetraploid mouse decidual cells was taken from [34]. Polyploid and diploid cells were separated with the method of centrifugation. The genome-wide gene expression profile was assessed using microarray hybridization and analysis according to the Affymetrix recommended protocols [34]. The raw data has been deposited at the GEO website (accession number GSE28917).

(4) One more database contains data related to tetraploid and diploid human trophoblastic MSC [35]. In this study, human extravillous trophoblastic diploid and polyploid cells were separated using flow cytometry with a BD FACS Canto II Cell Analyzer. The gene expression profile in diploid and tetraploid cells was evaluated with mRNA sequencing on a NovaSeq 6000 S1Flow Cell (Illumina) system. The datasets presented in this study can be found at BioProject (accession number PRJNA724881) and at the GEO website (accession number GSE173372).

The mRNA seq data for CP and young CARD obtained from iPC of the patients with HPLV and of healthy controls were taken from [36]. Here, we focused on CP obtained on the second day after the induction of cardiogenic differentiation in iPC from patients with HPLV and on the young cardiomyocytes (CARD) obtained on day 12 from the same iPC (bulk RNA sequencing data were obtained from #GSE135411). From these data, the amount of CP and young CARD with polyploid nuclei and several nuclei was several folds higher in the cells from the HPLV patients compared to health control [36]. The evaluation of cell ploidy in this study was performed using Flow cytometry.

### 4.2. Obtaining the Sets of Ploidy-Induced and Ploidy-Suppressed Consensus Genes

First of all, we identified differentially expressed genes (DEGs) between polyploid and diploid cells. The transcript levels (called ‘expression’ for brevity) were normalized uniformly for all datasets using the limma’ software https://bioconductor.org/packages/release/bioc/html/limma.html (accessed on 20 June 2023) implemented in the R package (with the quantile normalization method) [127]. The limma is the most universal approach for disparate datasets; it can treat both natural (counts) and real numbers. The revealing of DEGs was also performed using the limma software. The DEGs were selected according to the following criteria: *p*-value < 0.05 and adjusted *p*-value (FDR) < 0.1. Then, we selected the genes that were orthologous between human and mouse using the NCBI orthologs database (https://www.ncbi.nlm.nih.gov, accessed on 20 June 2023). In order to maximize the accuracy of identifying common manifestations of polyploidy in MSC and tumor cells, we focused exclusively on the “consensus” genes. These are the genes that were consistently induced or suppressed in all five polyploid vs. diploid comparisons taken from the databases for normal MSC and tumors. Genes induced and suppressed by polyploidy were analyzed separately. Only the statistically significant DEGs were included in the analysis. As a result, 358 induced and 422 suppressed consensus genes were selected. Because these databases are independent, the overall statistical significance for consensus DEGs is much higher (probabilities of independent events are multiplied).

### 4.3. Obtaining the Sets of Ploidy-Induced or Ploidy-Suppressed Master Regulator Genes

To reveal the polyploidy-associated master regulator genes, we applied the protein interaction enrichment analysis (PIEA) to ploidy-regulated genes with more than 5 interactants. The PIEA was performed according to [37]. In brief, PIEA determines the enrichment of protein interactants from the one-step neighborhood of the protein encoded by a given gene in a tested gene set. This procedure is similar to the enrichment analysis of pathways, processes, and other gene signatures in a tested gene set. Yet, as a gene signature, the set of one-step interactants of a given protein is used. In other words, PIEA identifies the most important master regulators, whose one-step interactome neighborhood is modularly enriched in a given DEG set. In our case, these are the hub genes whose interactome was most strongly up- or downregulated with the change of ploidy. It is important to note that the identification of hub regulators increases the sensitivity and robustness of the method and therefore is particularly appropriate for the identification of subtle effects with high robustness [128].

### 4.4. Enrichment Analysis of All Consensus DEGs and Consensus Master Regulators Associated with Polyploidy

For the enriched gene modules and signatures for polyploid vs. diploid MSC and tumor cells, we used the Metascape (https://metascape.org/gp/index.html#/main/step1 accessed on 18 September 2023) [41]. This is a web-based portal for comprehensive gene annotation and analysis that covers over 40 independent knowledge bases in an integrated database, including functional enrichment, interactome analysis, gene annotation, and a membership search in combination with Gene Ontology (GO) and the Kyoto Encyclopedia of Genes and Genomes (KEGG) tools [41]. Moreover, Metascape provides the gene set analysis in the context of protein interactions and applies a molecular complex identification algorithm MCODE to extract protein complexes embedded in the large networks [41,129].

To verify the results obtained with Metascape and obtain new evidence, we compiled an extensive collection of gene signatures and gene modules, encompassing gene sets from various databases (BioSystems, KEGG, Reactome, Canonical Pathways, WikiPathways, Molecular Signatures Database, Pathway Interaction Database, and Tumor Suppressor Gene database (TSGene https://bioinfo.uth.edu/TSGene accessed on 9 September 2023)), the Catalogue of somatic mutations in cancer (COSMIC), Network of Cancer Genes (NCG), EpiFactors database, AnimalTFDB (animal transcription factors), HisgAtlas (human immunosuppression gene database), etc., as well as those published in the literature [37,44,45,47,48,49,50,130,131,132,133,134,135].

The analysis of enriched signatures was conducted for all consensus DEGs and consensus master regulators associated with polyploidy. The total gene set from the human genome was used as a background for analysis. The enriched signatures with FDR-values < 0.01 and O/E ratio > 1.5 were collected. (The O/E ratio is the ratio of the number of observed genes from a signature to the number of expected genes based on a random distribution.) Specifically, p-values were calculated on the basis of cumulative hypergeometric distribution, using the Benjamini–Hochberg procedure for multiple testing corrections to obtain FDR values. In addition, we analyzed gene composition for essential pathways with unclear functions, i.e., pathways implicated in cancer and cell activation, using the String server [42].

## 5. Conclusions

Altogether, our results present the first comprehensive characterization of common and evolutionarily conserved mechanisms of ploidy-associated epigenetic regulation of the transcriptome of cancer cells and MSC. The main result reveals a functionally cohesive picture of ploidy-related changes that are coordinated by the activation of the NUA4/Tip60 chromatin remodeling complex operating via histone acetylation. Many biological functions of this complex, including canonical (chromatin opening and regulation of DNA Damage Response) and moon light (ciliogenesis, regulation of centrosome cycle, and chromosome segregation in mitosis) functions, are associated with polyploidy. The other ploidy-associated traits can also originate from NUA4/Tip60 hyperactivity. Thus, chromatin opening promotes hypertranscription, stemness, and unicellularity; NUA4/Tip60-associated DNA damage response activates c-Myc and further chromatin opening; and the activation of ciliogenesis increases resistance to apoptosis and drugs, impairs immunity and circadian clocks, and boosts signaling of multipotency. The presented results expand our knowledge of the role of polyploidy in the epigenetic regulation of gene expression and help to elucidate why polyploidy activates certain biological processes while suppressing others. Furthermore, our data suggests that genome duplications can generate global epigenetic changes leading to the increased complexity of gene regulatory networks thereby enabling the combination of programs that are incompatible in diploid state (e.g., the programs of stemness and differentiation as well as evolutionary ancient and young programs). Importantly, our data open the way for future research exploring the effects of polyploidy in various biological contexts, including in disease progression and therapeutic interventions. The obtained data can also be valuable for therapeutic practices in multiple directions: (1) they will contribute to the development and implementation of approaches enhancing the therapeutic properties of MSC by removing polyploid cells; (2) they will assist in the creation of new approaches in targeted therapy for cancer cells using a combination of multiple strategies, including the suppression of signaling cascades implicated in proliferation, unicellularity, and ciliogenesis; and (3) they will aid in the development of new approaches for the epigenetic correction of cancer cells.

## Figures and Tables

**Table 1:** Gene signatures and pathways that are enriched in ploidy upregulated usual genes (358 gene set) and for master regulators (113 gene set).

Pathway/Signature	Gene Number	O/E Ratio *	FDR **
**Housekeeping functions**			
Organelle biogenesis and maintenance (Reactome)	16	8.18	4.78 × 10^−08^
Housekeeping genes [46] #&	50	2.34	2.02 × 10^−07^
Gene Expression (Reactome)	33	3.17	3.64 × 10^−07^
TF-cofactors Animal TFDB [47]	23	3.88	3.58 × 10^−06^
Intra-Golgi and retrograde Golgi-to-ER traffic (Reactome)	10	9.62	1.66 × 10^−05^
Golgi-to-ER retrograde transport (Reactome)	7	11.00	4.43 × 10^−04^
AURKA Activation by TPX2 (Reactome)	6	14.08	5.24 × 10^−04^
Centrosome maturation; Recruitment of mitotic centrosome proteins and complexes (Reactome) #&	6	12.85	7.83 × 10^−04^
COPI-dependent Golgi-to-ER retrograde traffic (Reactome) #	6	12.69	8.16 × 10^−04^
Regulation of PLK1 Activity at G2/M Transition (Reactome) #&	6	11.68	1.21 × 10^−03^
IGF1 pathway (Pathway Interaction Database) #&	4	24.47	1.82 × 10^−03^
Top 10% AT3 SW-degenerate synonymous sites [37] #&	27	2.33	1.88 × 10^−03^
3rd PIN cluster [45] #&	22	2.62	2.28 × 10^−03^
Membrane Trafficking (Reactome)	13	3.67	4.33 × 10^−03^
Processing of Capped Intron-Containing Pre-mRNA (Reactome)	8	5.76	5.96 × 10^−03^
mRNA Splicing—Major Pathway (Reactome)	7	6.74	6.12 × 10^−03^
Mitotic G2-G2/M phases (Reactome)	7	6.66	6.30 × 10^−03^
Vesicle-mediated transport (Reactome)	13	3.45	6.62 × 10^−03^
Insulin-like Growth Factor-2 mRNA Binding Proteins (IGF2BPs/IMPs/VICKZs) bind RNA (Reactome)	2	114.18	6.62 × 10^−03^
mRNA Splicing (Reactome) #&	7	6.45	7.14 × 10^−03^
Spliceosome (Kegg)	6	7.73	8.24 × 10^−03^
Top 10% selection-favored in mammals [44]	21	2.38	9.03 × 10^−03^
Cell Cycle, Mitotic (Reactome)	11	3.71	1.15 × 10^−02^
Cell Cycle (Reactome)	12	3.35	1.45 × 10^−02^
Cellular responses to stress (Reactome)	9	3.47	5.77 × 10^−02^
RNA Polymerase II Transcription (Reactome)	5	5.72	8.24 × 10^−02^
Insulin Pathway (Pathway Interaction Database) #&	3	11.95	8.60 × 10^−02^
**Unicellularity**			
Unicellular genes [48] #	77	2.08	1.30 × 10^−11^
Human-yeast 1:1 orthologs (Ensembl)	19	3.37	4.28 × 10^−04^
Unicellular genes [49] #&	52	1.75	6.14 × 10^−04^
Unicellular PIN cluster [44]	36	1.94	3.26 × 10^−03^
**Myc signaling and stemness**			
MYC interactants (String)	15	3.18	5.61 × 10^−03^
Kit receptor signaling pathway (Reactome)	4	2.18	5.48 × 10^−03^
HALLMARK_MYC_TARGETS (Molecular Signatures Database) #	7	5.99	1.06 × 10^−02^
C-MYC pathway (Pathway Interaction Database) #&	3	23.35	1.57 × 10^−02^
**Chromatin and DNA damage response**			
EpiFactors database [50] #&	26	6.23	2.73 × 10^−11^
Chromatin organization; Chromatin modifying enzymes (Reactome) #&	14	8.72	2.41 × 10^−07^
HATs acetylate histones (Reactome) #&	8	9.65	2.87 × 10^−04^
HALLMARK_DNA_REPAIR (Molecular Signatures Database) #	8	9.13	3.96 × 10^−04^
DNA Damage/Telomere Stress Induced Senescence (Reactome) #	4	8.56	5.95 × 10^−02^
Epigenetic regulation of gene expression (Reactome) #	5	5.79	8.05 × 10^−02^
**Ciliogenesis**			
Cilium Assembly (Reactome) #&	14	12.75	2.39 × 10^−09^
Anchoring of the basal body to the plasma membrane (Reactome) #	9	15.73	1.43 × 10^−06^
Loss of proteins required for interphase microtubule organization from the centrosome; Loss of Nlp from mitotic centrosomes (Reactome) #	6	14.68	4.41 × 10^−04^

* O/E (Observed/Expected) ratio: the ratio of the number of observed genes to the number of genes expected under a random distribution. ** FDR (false discovery rate): statistical significance adjusted for multiple testing. #—Confirmed by CP; &—Confirmed by young CARD. Gene pathways and signatures from the same functional groups are marked with similar colors.

**Table 2:** Gene signatures and pathways that are enriched in ploidy downregulated genes (425 gene set) and for master regulators (105 gene set).

Pathway/Signature	Gene Number	O/E * Ratio	FDR **
**Immunity**			
HALLMARK_TNFA_SIGNALING_VIA_NFKB (Molecular Signatures Database)	18	16.11	2.03 × 10^−14^
Immune System (Reactome)	39	3.36	7.21 × 10^−10^
HALLMARK_INFLAMMATORY_RESPONSE (Molecular Signatures Database)	14	12.53	1.39 × 10^−09^
Regulation of toll-like receptor signaling pathway (WikiPathways)	11	14.17	5.53 × 10^−08^
Cytokine Signaling in Immune system (Reactome)	21	5.07	1.12 × 10^−07^
Innate Immune System (Reactome)	26	3.67	6.75 × 10^−07^
Toll-like Receptor Signaling Pathway (WikiPathways)	8	14.04	1.07 × 10^−05^
Toll-like receptor signaling pathway (Kegg)	8	13.77	1.21 × 10^−05^
Signaling by Interleukins (Reactome)	15	5.21	1.62 × 10^−05^
Thymic Stromal LymphoPoietin (TSLP) Signaling Pathway (WikiPathways)	6	22.86	2.01 × 10^−05^
IL2-mediated signaling events (Pathway Interaction Database)	6	20.66	3.22 × 10^−05^
HALLMARK_IL2_STAT5_SIGNALING (Molecular Signatures Database)	9	8.10	1.11 × 10^−04^
Toll-Like Receptors Cascades (Reactome)	8	9.55	1.17 × 10^−04^
TNF related weak inducer of apoptosis (TWEAK) Signaling Pathway (WikiPathways)	5	21.32	2.07 × 10^−04^
DAP12 signaling (Reactome)	11	5.60	2.54 × 10^−04^
DAP12 interactions (Reactome)	11	5.37	3.65 × 10^−04^
HALLMARK_ALLOGRAFT_REJECTION (Molecular Signatures Database)	10	8.95	1.62 × 10^−05^
TNF signaling pathway (Kegg)	6	9.95	1.41 × 10^−03^
Fc epsilon receptor (FCERI) signaling (Reactome)	10	4.87	1.67 × 10^−03^
Activated TLR4 signaling (Reactome)	6	9.51	1.67 × 10^−03^
**Apoptosis and cell death**			
Apoptosis—multiple species (Kegg)	9	50.36	2.96 × 10^−11^
Apoptosis Modulation and Signaling (WikiPathways)	10	19.68	1.54 × 10^−08^
Apoptosis (Kegg)	10	12.98	6.75 × 10^−07^
Programmed Cell Death (Reactome)	10	10.47	4.72 × 10^−06^
Intrinsic Pathway for Apoptosis (Reactome)	6	24.98	1.33 × 10^−05^
HALLMARK_APOPTOSIS (Molecular Signatures Database)	9	10.01	2.48 × 10^−05^
Apoptosis (Reactome)	9	9.59	3.22 × 10^−05^
Apoptosis (WikiPathways)	7	14.92	3.22 × 10^−05^
Apoptosis-related network due to altered Notch3 in ovarian cancer (WikiPathways)	6	20.27	3.48 × 10^−05^
Caspase cascade in apoptosis (Pathway Interaction Database)	5	17.91	4.58 × 10^−04^
**Circadian clock**	9	35.03	8.55 × 10^−10^
BMAL1:CLOCK, NPAS2 activates circadian gene expression (Reactome)	9	35.03	8.55 × 10^−10^
Circadian Clock (Reactome)	9	23.35	2.77 × 10^−08^
Circadian rhythm related genes (WikiPathways)	12	10.69	1.91 × 10^−07^
Circadian rhythm (Kegg)	5	28.88	5.12 × 10^−05^
Circadian rhythm related genes (WikiPathways)	12	10.69	1.91 × 10^−07^
**Tumor suppressor genes**	23	4.22	4.85 × 10^−07^
TSGene downregulated pancancer (Tumor Suppressor Gene database	23	4.22	4.85 × 10^−07^
TSGene all (Tumor Suppressor Gene database)	23	4.06	9.07 × 10^−07^
Validated targets of C-MYC transcriptional repression (Kegg)	5	11.74	8.97 × 10^−04^
Hippo signaling pathway (Kegg)	12	5.45	6.05 × 10^−03^

* O/E (Observed/Expected) ratio: the ratio of the number of observed genes to the number of genes expected under a random distribution. ** FDR (false discovery rate): statistical significance adjusted for multiple testing. Gene pathways and signatures from the same functional groups are marked with similar colors.

## Data Availability

Data are contained within the article and Supplementary Materials.

## Supplementary Materials

The following supporting information can be downloaded at: https://www.mdpi.com/article/10.3390/ijms25084185/s1.
