# Supplementary material for: Polyploidy Promotes Hypertranscription, Apoptosis Resistance, and Ciliogenesis in Cancer Cells and Mesenchymal Stem Cells of Various Origins: Comparative Transcriptome In Silico Study
Source: Int J Mol Sci. 2024 Apr 10;25(8):4185. doi: 10.3390/ijms25084185 (PMC11050069; doi:10.3390/ijms25084185)
Supplement: Supplementary file 1 [file ijms-25-04185-s001.zip › Supplementary Figures All.pdf]

Supplementary Figures

Supplementary Figure S1

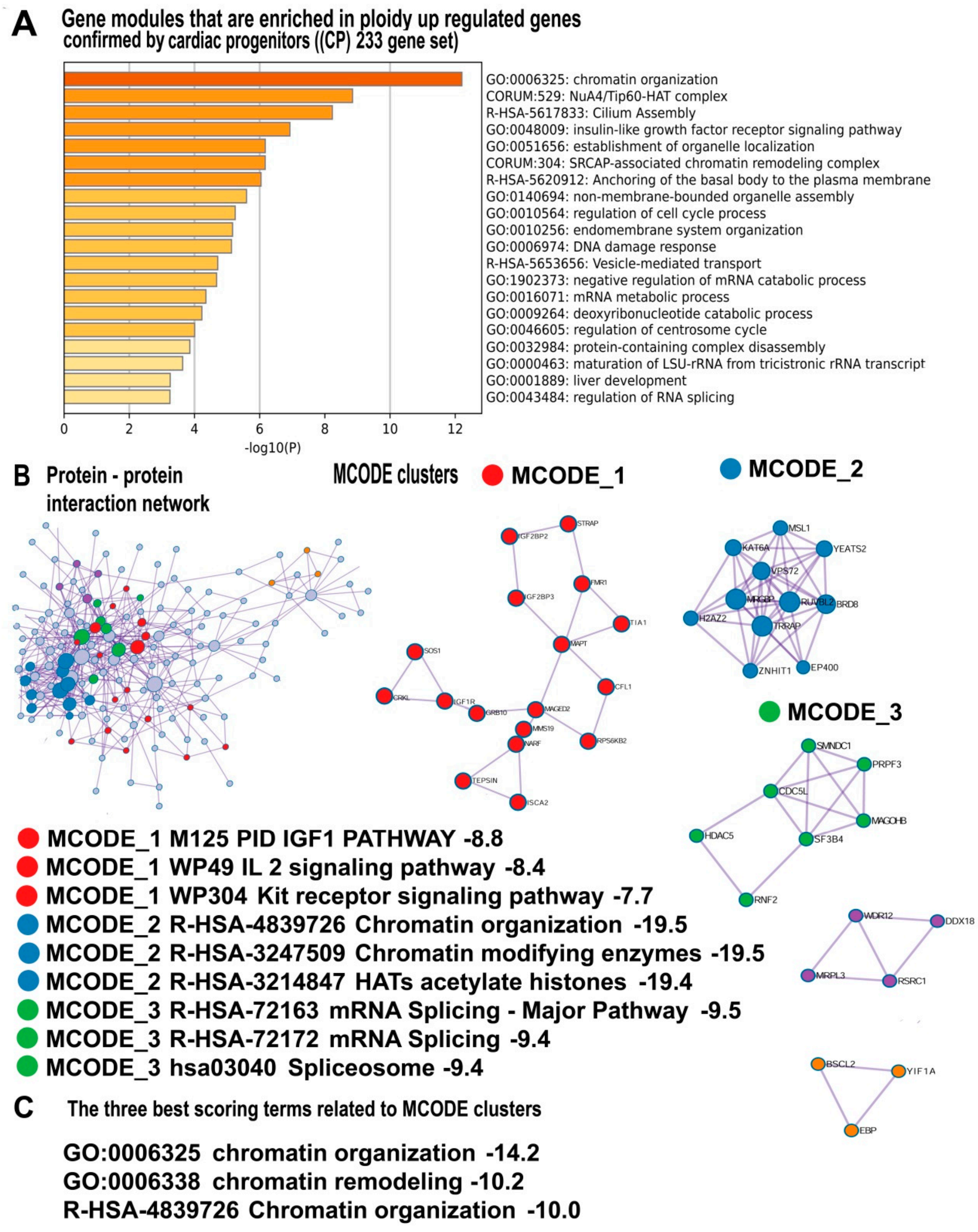

Supplementary Figure S1. The enrichment of polyploidy-induced genes from the 233 gene set for CP. This gene set was obtained by comparison of ploidy-upregulated genes in cancer cells and MSC (358 gene set) and all ploidy-upregulated genes in cardiac progenitor cells (CP) obtained from iPS of fibroblastic origin **[28].** (A)—Bar graph of enriched terms related to gene modules and processes. The statistical significance of enrichment is shown at the X-axis (−log10 (p)). (**B**) — Protein interaction network and MCODE components (densely connected network components) identified in the gene list. The network and MCODE components were constructed on the base of physical interactions taken from String server (physical score > 0.4). The coding by color circles indicates the results of MCODE

component pathway and process enrichment analysis. (**C**) — The three best-scoring terms related to MCODE components. This Figure illustrates a good concordance between the data obtained for all 358 genes (for cancer cells and MSC) and for the 233 gene set (for CP derived from iPS). This is clearly seen for general chromatin remodeling (**A-C**) and chromatin remodeling associated with NuA4/Tip60 histone acetylating complex. It also uncovers the activation of housekeeping and developmental processes (A, B), and ciliogenesis (**A**).

Supplementary Figure S2

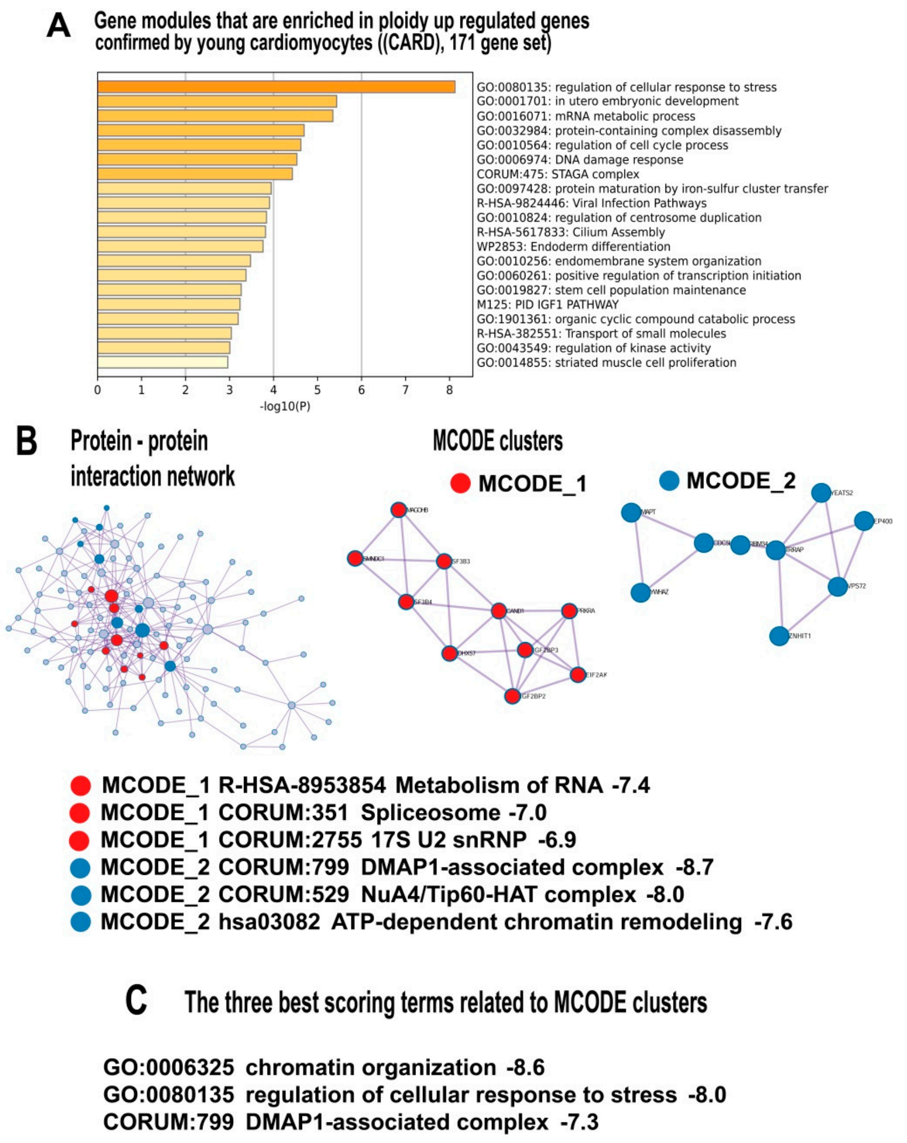

Supplementary Figure S2. The enrichment of polyploidy-induced genes from the 171 gene set for young CARD . This gene set was obtained by comparison of ploidy-upregulated genes in cancer cells and MSC (358 gene set) and all ploidy-upregulated genes in young cardiomyocytes (CARD) obtained from iPS of fibroblastic origin **[28].** (A)—Bar graph of enriched terms related to gene modules and processes. The statistical significance of enrichment is shown at the X-axis (−log10 (p)). (**B**) — Protein interaction network and MCODE components (densely connected network components) identified in the gene list. The network and MCODE components were constructed on the base of physical interactions taken from String server (physical score > 0.4). The coding by color circles indicates the results of MCODE component pathway and process enrichment analysis. (**C**) — The three best-scoring terms

related to MCODE components. This Figure illustrates a good concordance between the data obtained for all 358 genes (for cancer cells and MSC) and for the 171 gene set (for CP derived from iPS). This is clearly seen for general chromatin remodeling (**A-C**) and chromatin remodeling associated with NuA4/Tip60 histone acetylating complex. It also uncovers the activation of housekeeping and developmental processes, DNA damage response (A, B), and ciliogenesis (**A**).

Supplementary Figure S3

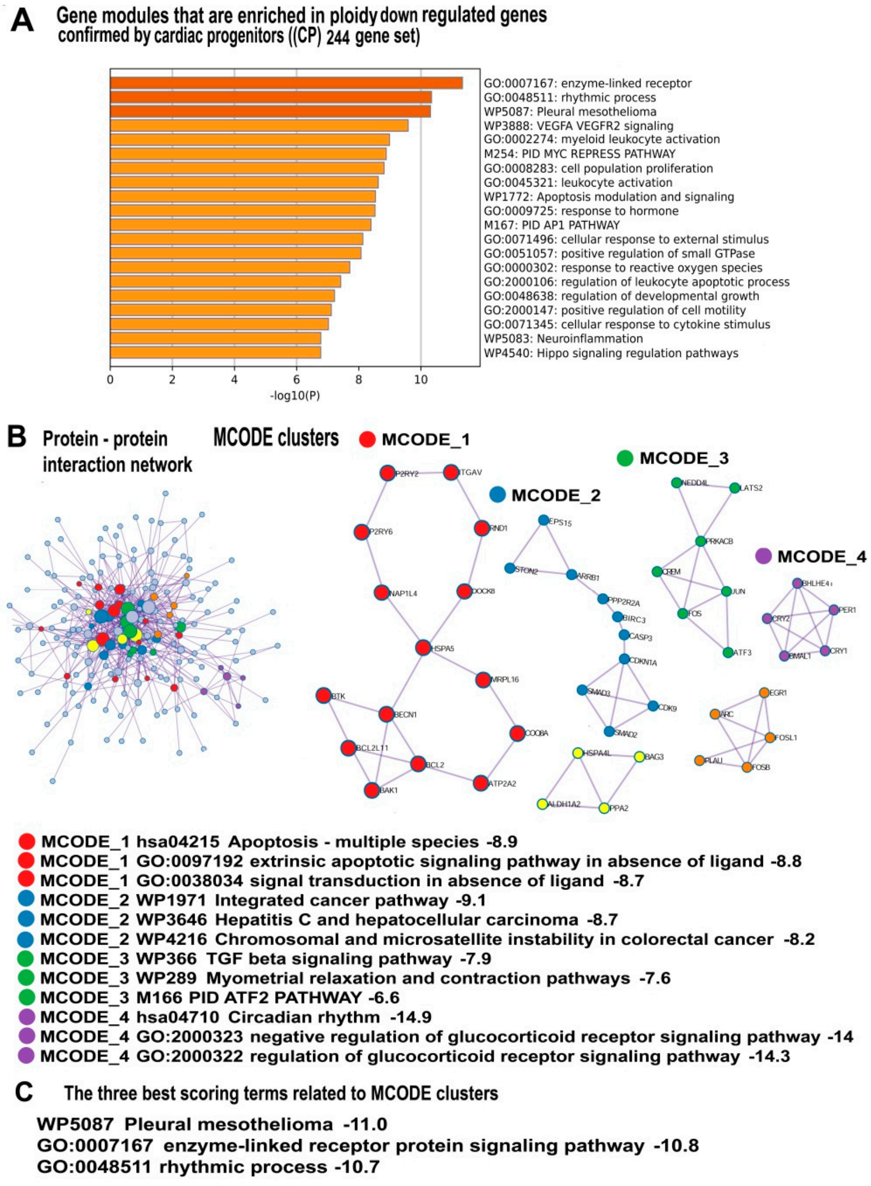

Supplementary Figure S3. The enrichment of polyploidy- downregulated genes from the 244 gene set. This gene set was obtained by comparison of ploidy-downgulated genes in cancer cells and MSC (425 gene set) and all ploidy-downregulated genes in cardiac progenitor cells (CP) obtained from iPS of fibroblastic origin **[28].** The enrichment of polyploidy-inhibited genes from the 244 gene set . (A)—Bar graph of enriched terms related to gene modules and processes. The statistical significance of enrichment is shown at the X-axis (−log10 (p)). (**B**) — Protein interaction network and MCODE components (densely connected network components) that were identified in the gene list. The network and MCODE components were constructed on the base of physical interactions taken from String server (physical score > 0.4). The coding by color circles indicates the results of MCODE component pathway and process enrichment analysis. (**C**) — Three best-scoring terms related to MCODE components. This Figure illustrates a good concordance between the data obtained for all 425 genes and 244 gene set. Especially, it is seen for the gene modules implicated in apoptosis, immunity (A-C), and circadian clock (A, B).

Supplementary Figure S4

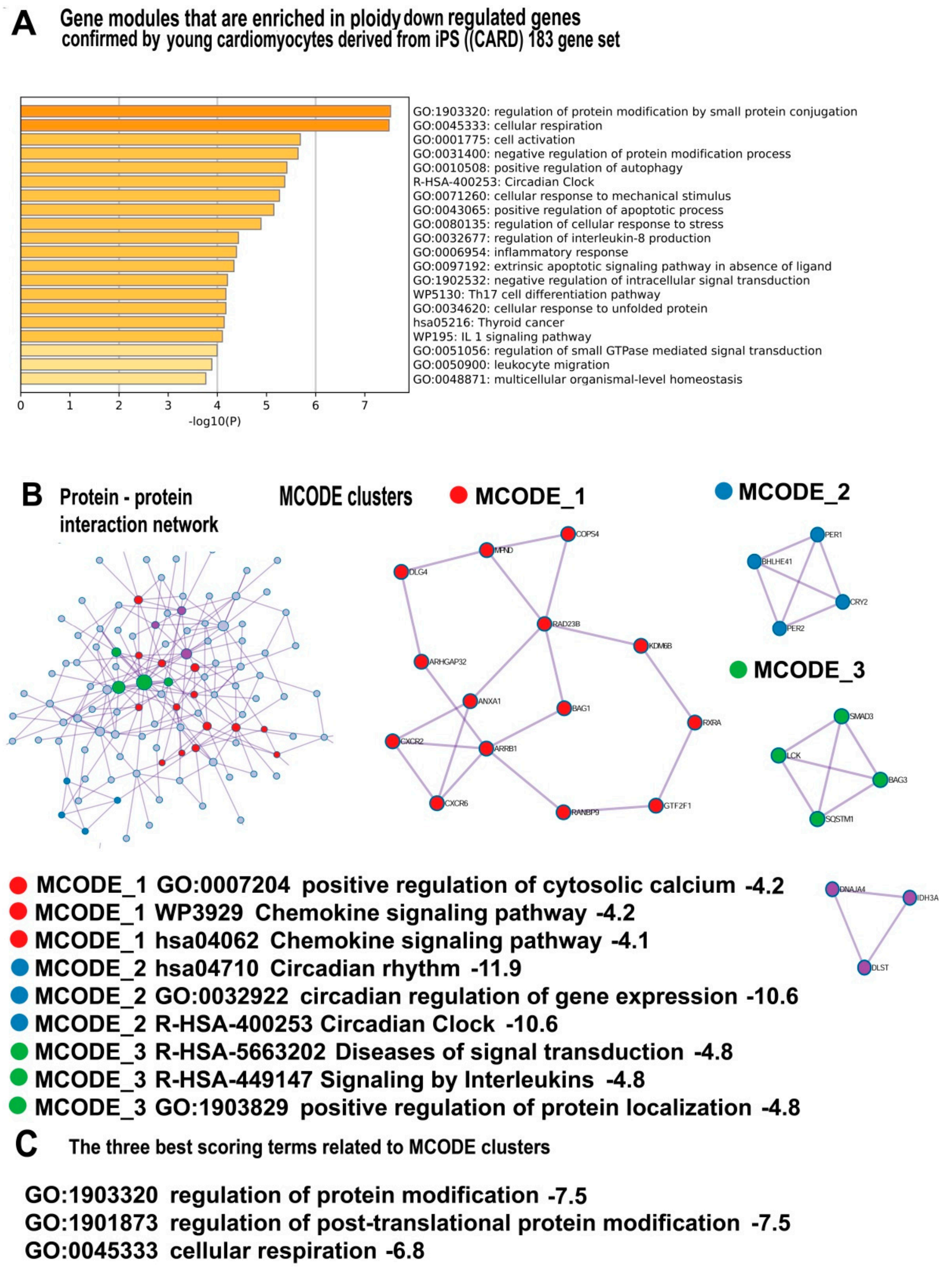

Supplementary Figure S4. The enrichment of polyploidy- downregulated genes from the 183 gene set. This gene set was obtained by comparison of ploidy-downgulated genes in cancer cells and MSC (425 gene set) and all ploidy-downregulated genes in young cardiomyocyte (CARD) obtained from iPS of fibroblastic origin **[28].** The enrichment of polyploidy-inhibited genes from the 183 gene set . (A)—Bar graph of enriched terms related to gene modules and processes. The statistical significance of enrichment is shown at the X-axis (−log10 (p)). (**B**) — Protein interaction network and MCODE components (densely connected network components) that were identified in the gene list. The network and MCODE components were constructed on the base of physical interactions taken from String server (physical score > 0.4). The coding by color circles indicates the results of MCODE component pathway and process enrichment analysis. (**C**) — Three best-scoring terms related to MCODE components. This Figure illustrates a good concordance between the data obtained for all 425 genes and 183 gene set. Specifically, it is seen for the gene modules implicated in apoptosis, immunity (A-C), and circadian clock (A, B).
